# Supplementary material for: An epigenome atlas of neural progenitors within the embryonic mouse forebrain
Source: Nat Commun. 2022 Jul 20;13:4196. doi: 10.1038/s41467-022-31793-4 (PMC9300614; doi:10.1038/s41467-022-31793-4)
Supplement: Supplementary file 1 — Supplementary Information [file 41467_2022_31793_MOESM1_ESM.pdf]

SUPPLEMENTARY FIGURES AND LEGENDS

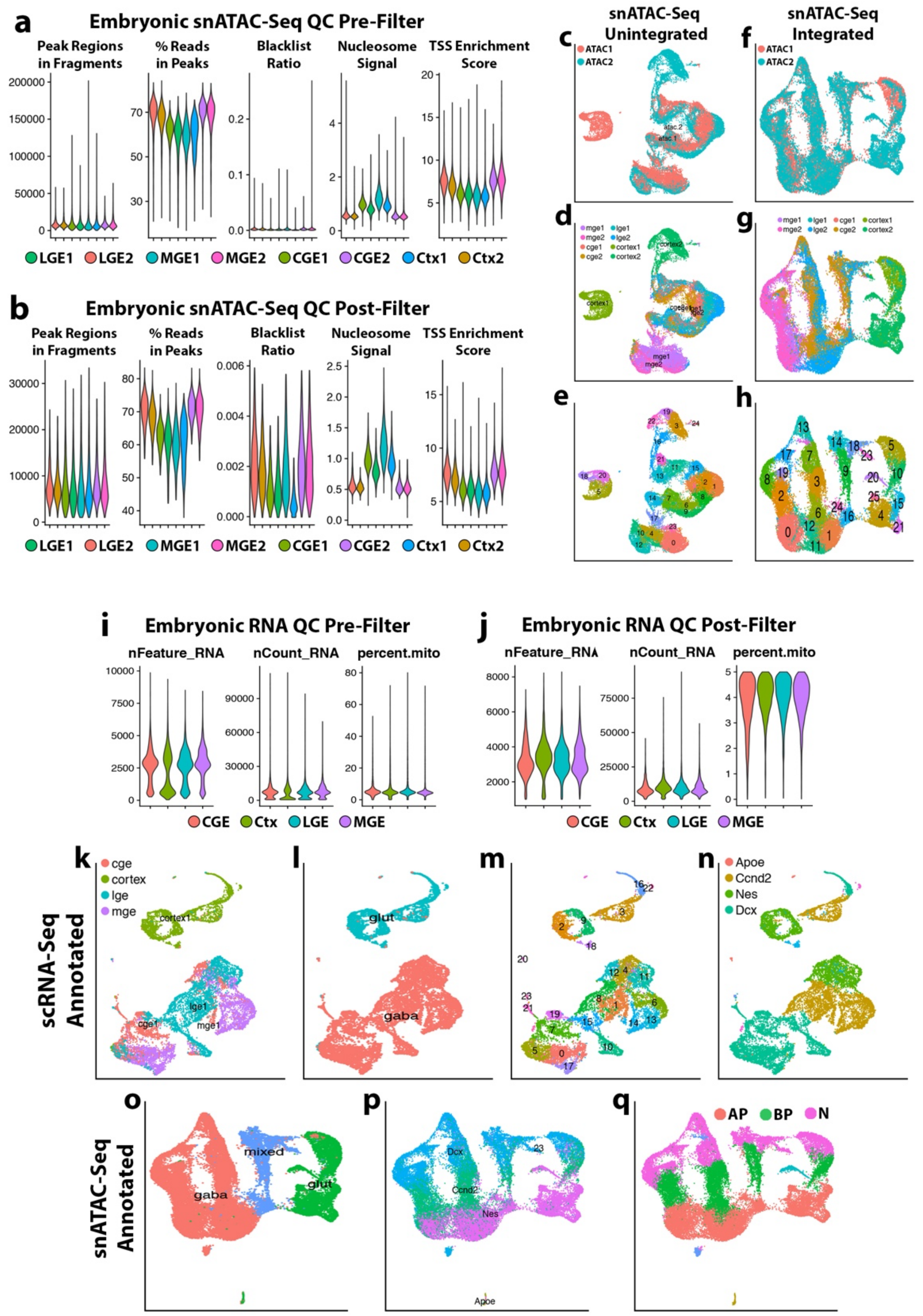

Supplementary Fig 1. snATAC-Seq and scRNA-Seq quality control and batch reduction, and scRNA-Seq based snATAC-Seq annotation. a, Violin plots of snATAC-Seq QC metrics before outlier removal. **b**, Violin plots of snATAC-Seq QC metrics after outlier removal. **c-e**, UMAP visualization of snATAC-Seq nuclei prior to batch removal colored by replicate (**c**), tissue origin (**d**) and SLM cluster (**e**). **f-h**, UMAP visualization of batch corrected snATAC-Seq nuclei colored by replicate (**f**), tissue origin (**g**) and SLM cluster (**h**). **i**, Violin plots of scRNA-Seq QC metrics before outlier removal. **j**, Violin plots of scRNA -Seq QC metrics after outlier removal. **k-n**, UMAP visualization of scRNA-Seq cells colored by tissue (**k**), neuronal class (**l**), Louvain cluster (**m**) and cell type (**n**). **o-q**, UMAP visualization of snATAC-Seq nuclei colored by neuronal class (**o**), scRNA-Seq cell type predictions (**p**) and assigned neurogenic cell type (**q**).

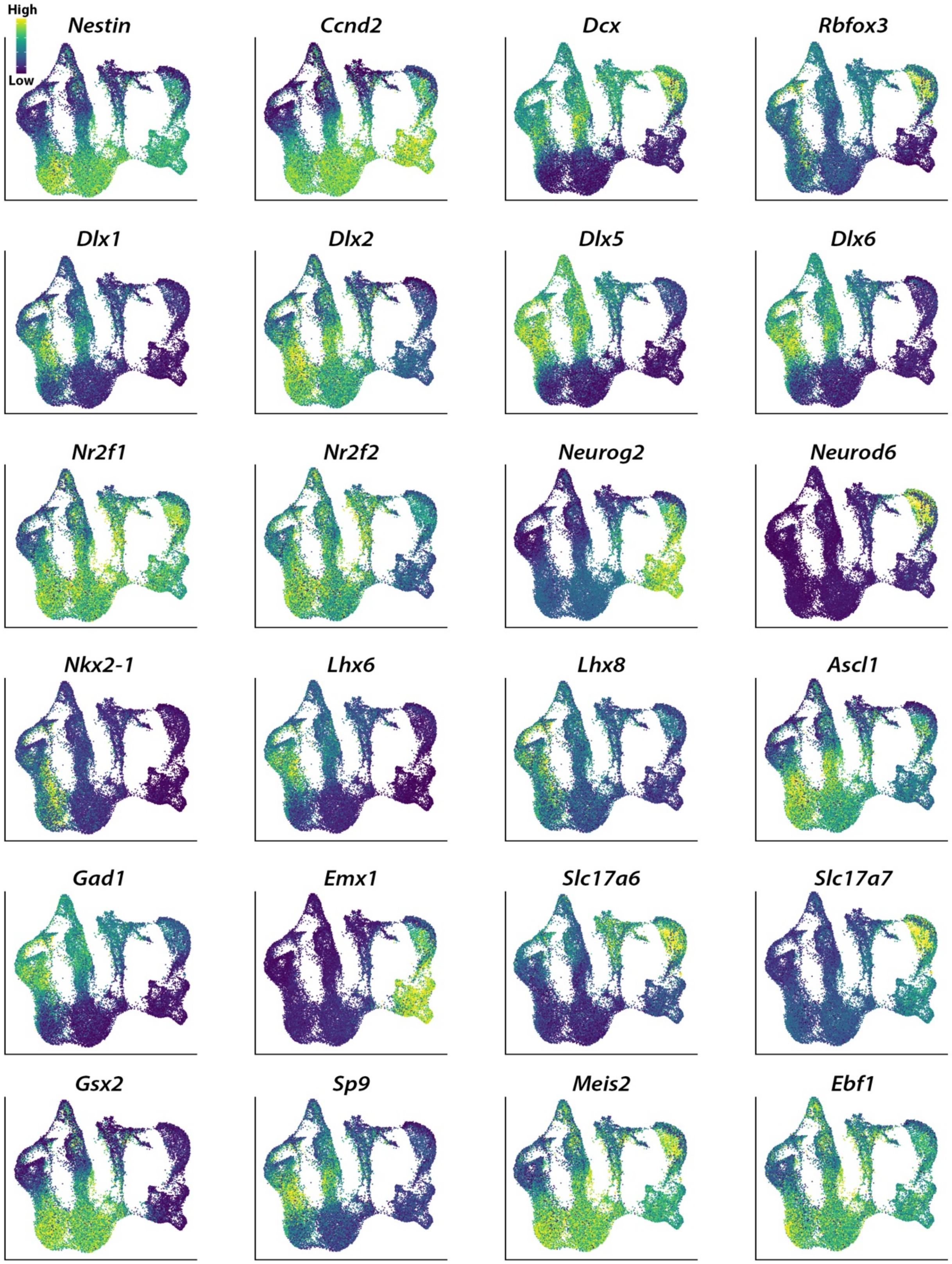

Supplementary Fig. 2. Visualization of GAS for critical interneuron markers. UMAP visualization of GAS from snATAC-Seq nuclei for specified genes. Legend depicts normalized read counts.

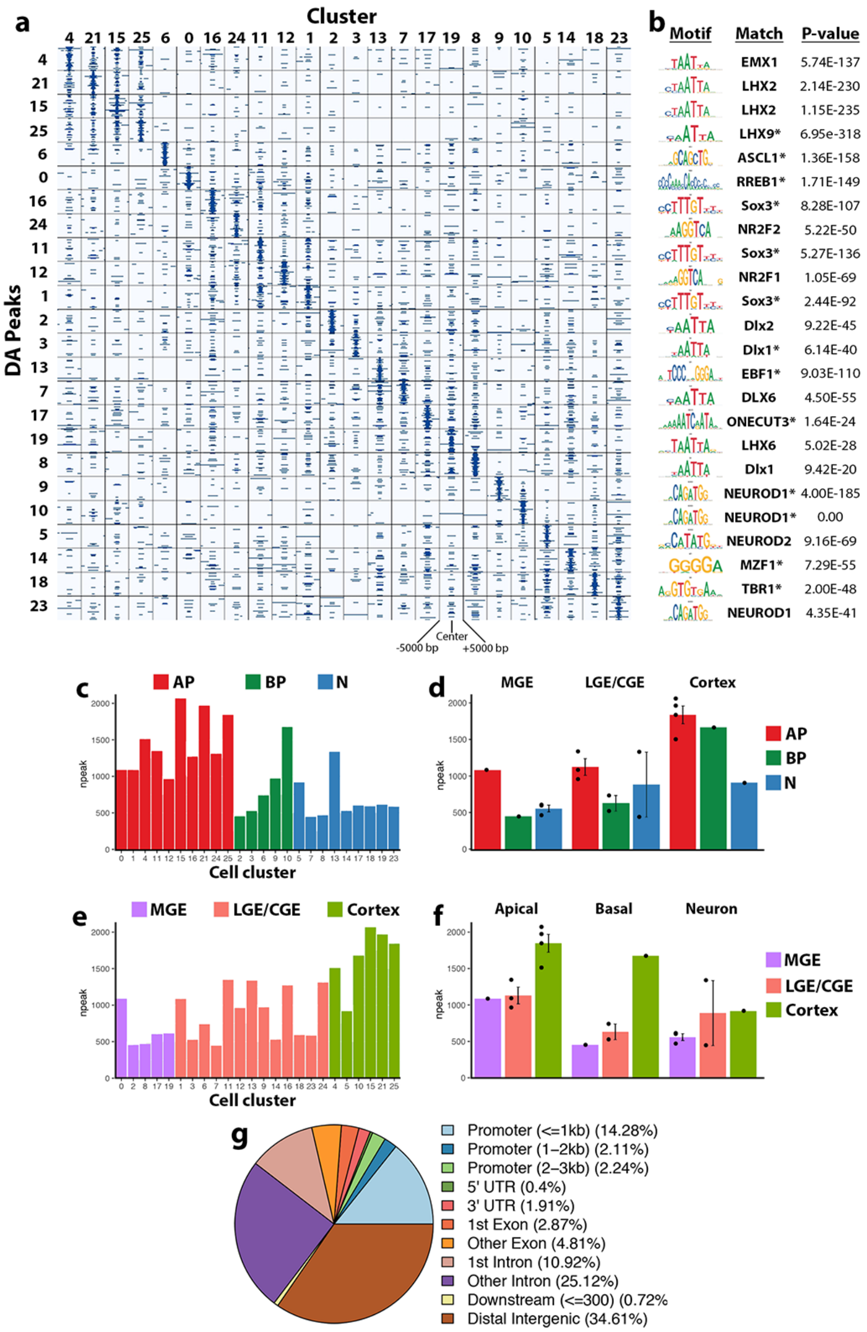

Supplementary Fig. 3. DA Peaks per cluster and read distribution across gene features. **a**, Embryonic snATAC-Seq signal plots of DA peaks for each cluster (same cluster order as Fig 2). Signal was binarized to indicate DA peaks from one cluster (column cluster labels) which are detectable in any other cluster (row cluster labels). **b**, For each cluster (rows of the dot plot), motifs representative of lineage and maturation stage, corresponding motif name and P-value are displayed to the right of the dot plot. All motifs are in the top five hits for each cluster, with top hits indicated with an asterisk. **c**, Bar chart of DA peak counts where -log(Fold Change) > 0 per SLM cluster colored by neurogenic cell type. **d**, Bar graph of mean DA peak counts where -log(Fold Change) > 0 per SLM cluster colored by neurogenic cell type and grouped by tissue. **e**, Bar chart of DA peak counts where -log(Fold Change) > 0 per SLM cluster colored by tissue. **f**, Bar chart of mean DA peak counts where -log(Fold Change) > 0 per SLM cluster colored by tissue and grouped by neurogenic cell type. **g**, Pie chart of relative enrichment of DA peaks within annotated gene features. For **d** and **f**, the n (number of clusters) in each group is as follows: MGE AP = 1, MGE BP = 1, MGE N = 3, LGE/CGE AP = 3, LGE/CGE BP = 2, LGE/CGE N =2, Cortex AP = 4, Cortex BP = 1, Cortex N = 1. For groups where n > 1, data are presented as mean values +/- SEM.

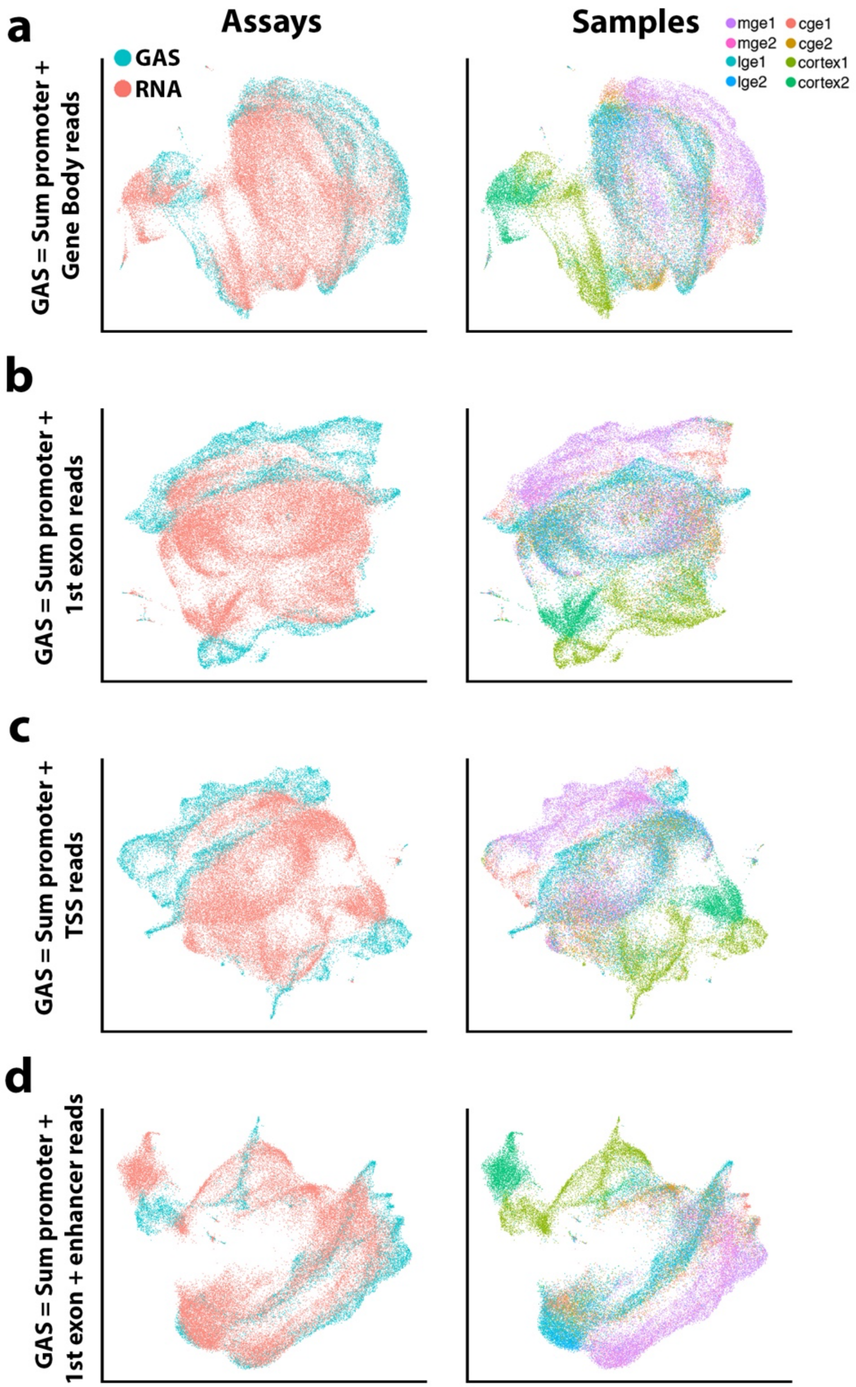

Supplementary Fig. 4. Gene activity score comparisons. UMAP visualizations of integrated snATAC-Seq and scRNA-Seq data colored by assay (left) and tissue samples (right). For integration, snATAC-Seq Gene Activity Score (GAS) was quantified using four different strategies: (**a**) quantified peaks within gene promoters (-2000 bp to 0 bp of 5' transcript coordinate) and gene bodies, (**b**) quantified peaks within gene promoters and the first exon of all transcripts, (**c**) quantified peaks within gene promoters only, and (**d**) quantified peaks within promoters, the first exon of all transcripts, and any associated putative enhancers (H3K27ac+ chromatin).

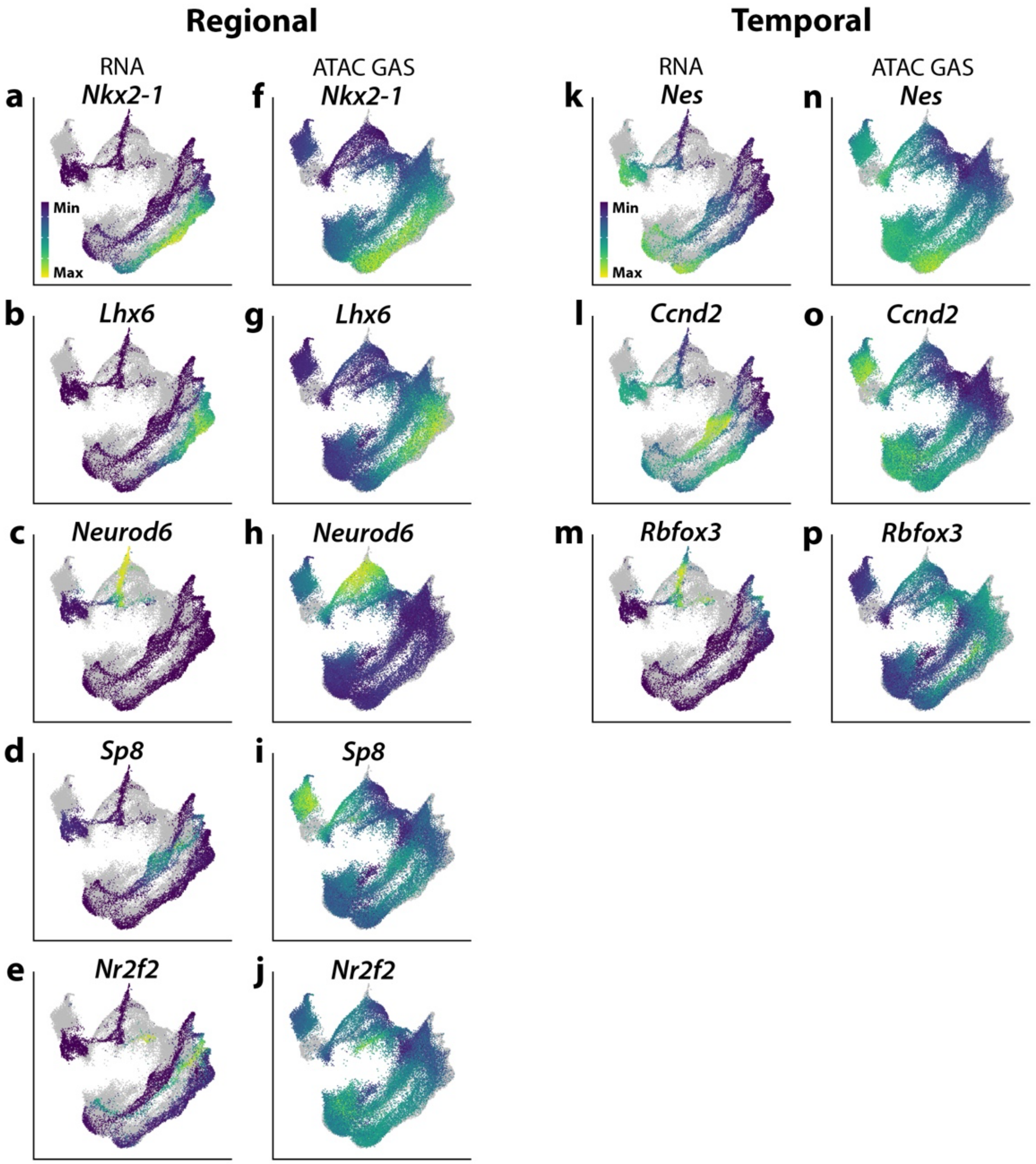

Supplementary Fig 5. Visualization of RNA counts and GAS following integration. UMAP visualization of transcript abundance from scRNA-Seq cells (left) and GAS from snATAC-seq nuclei (right) for genes enriched in specific brain regions (**a-j**) or in distinct neurogenic cell types (**k-p**).

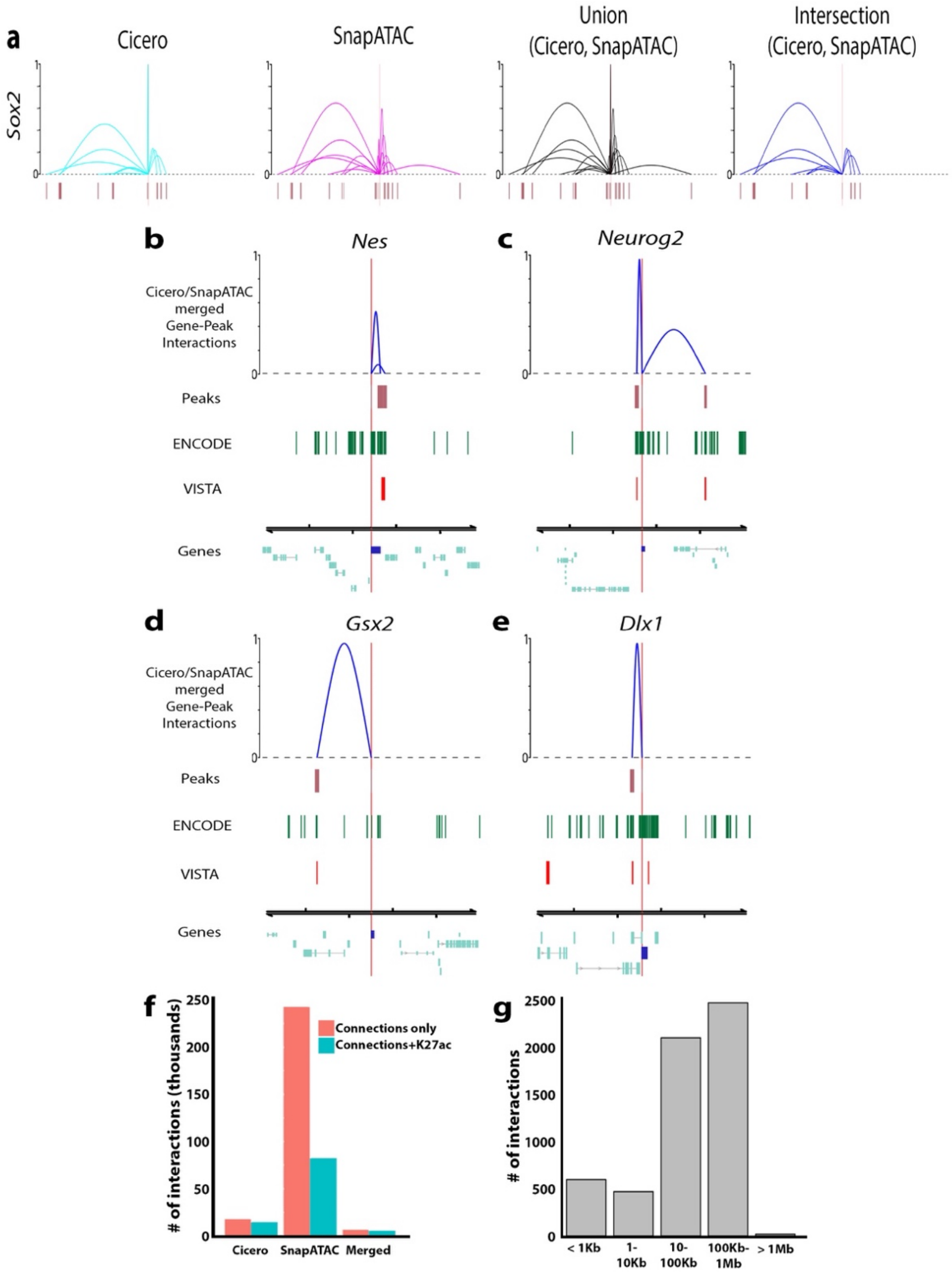

Supplementary Fig. 6. Characterization of enhancers in embryonic neurogenic tissue. a, Gene-enhancer interactions originating at the *Sox2* locus as defined by Cicero (cyan arcs), SnapATAC (magenta arcs), the combination of Cicero and SnapATAC (black arcs) and the intersection of Cicero and SnapATAC (dark blue arcs, representing high confidence enhancers). High confidence enhancers ("Intersection") were used for further analyses. Window is 0.5 Mb centered on TSS. Arc height represents interaction score. **b-e**, Visualization of intersection of Cicero and SnapATAC gene-peak interactions for *Nes* (**b**), *Neurog2* (**c**), *Gsx2* (**d**) and *Dlx1* (**e**). Window is 100 Kb, centered on the gene TSS. Peaks: displays co-accessible coordinates, Encode: displays H3K27ac signal from E12.5 forebrain from ENCODE ChIP-Seq data, VISTA: enhancers with positive activity in the forebrain and midbrain from the VISTA Enhancer Browser. **f**, Bar chart depicting the total number of gene-peak interactions detected by Cicero, SnapATAC and high-confidence interactions via the intersection of Cicero and SnapATAC ("merged" label). **G**, Bar chart of the number of gene-peak interactions binned by gene-peak distance for high-confidence gene-peak interactions.

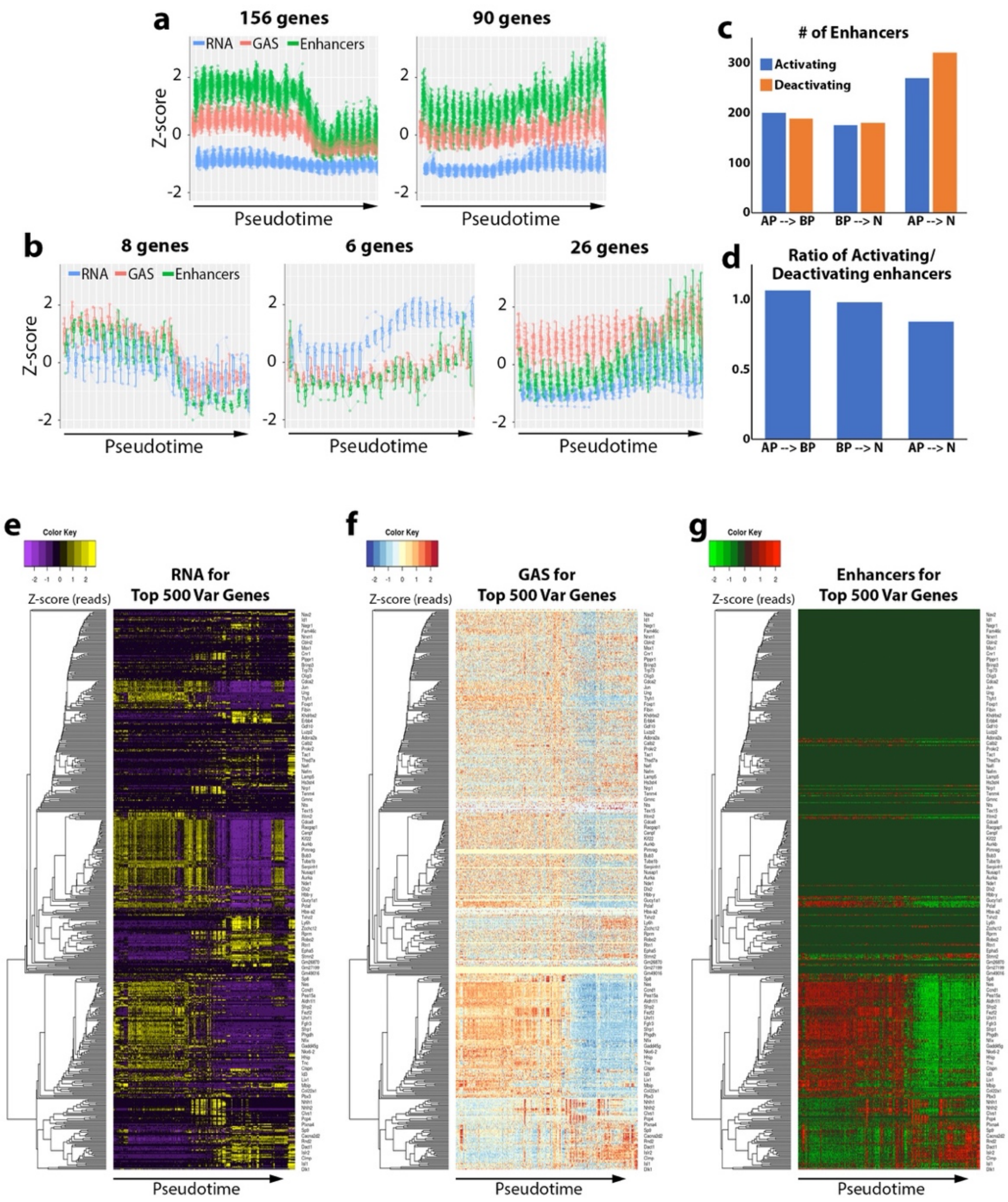

Supplementary Fig. 7. degPatterns analysis and heatmaps of RNA, GAS and enhancer counts for top 500 highly variable genes. a-b, Line graphs of genes within each cluster detected by degPatterns that contained > 5 genes per cluster using embryonic integrated snATAC-Seq/scRNA-Seq data. Y-axis is Z-score for RNA, GAS, or enhancers counts per gene. X-axis is binned pseudotime periods with origin corresponding to early development and higher pseudotime values corresponding to more differentiated cells. RNA, GAS, or enhancers for each gene in this cluster are plotted individually. The horizontal line in each box represents the median while the bottom and top edges represent the 1st and 3rd quartiles. The upper and lower whiskers extend from the edges of the box to no further than 1.5x of the inter-quartile range. **c**, Bar graph of change of enhancer usage for all detected H3K27ac+ presumptive enhancers associated with DEGs per state transition: AP to BP, BP to N and AP to N. All detectable H3K27ac+ presumptive enhancers defined as genomic intersection of 1) the genomic union of SnapATAC cREs and Cicero cREs with co-accessibility score >0.25) and 2) E12.5 ENCODE H3K27ac ChIP-Seq peaks. **d**, Bar graph of ratio of enhancer activation versus enhancer deactivation per state transition: AP to BP, BP to N and AP to N. **e-g**, Heatmaps of RNA (**e**), GAS (**f**) and enhancer (**g**) counts for top 500 highly variable genes from scRNA-Seq data and corresponding genes from snATAC-Seq GAS and enhancer matrices. Heatmap columns were ordered by hierarchical clustering with correlation distance and average linkage. Rows were ordered by pseudotime (assigned by Moncole3).

SUPPLEMENTARY DATA LEGENDS

Supplementary Data 1: Differentially accessible peaks per cluster
List of all differentially accessible peaks in each cell cluster that reached statistical significance. Adjusted p-value $\leq$ 0.05 (column E) based on a logistic regression and Bonferroni multi-test correction.

Supplementary Data 2: Differentially accessible motifs per cluster
List of all transcription factor binding motifs enriched in cluster-specific differentially accessible peaks that reached statistical significance. Adjusted p-value $\leq$ 0.05 (column J) based on the hypergeometric test and Bonferroni multi-test correction.

Supplementary Data 3: Differentially accessible motifs filtered by DEGs
List of top 5 transcription factor binding motifs in cluster-enriched peaks that correspond to differentially accessible promoters, grouped by brain region. Adjusted p-value $\leq$ 0.05 (column J) based on the hypergeometric test and Bonferroni multi-test correction.

Supplementary Data 4: Embryonic enhancers predicted by Cicero and SnapATAC
Combined co-accessibility analysis of Cicero and SnapATAC to determine candidate enhancers for specific genes in E12.5 and E14.5 tissue. Relevant statistics and metrics: Column G, Cicero Coaccesbility Score; Column H, Per gene percentile ranking of Cicero Coaccesbility and SnapATAC -log10(p-value) after adjusting to 1-100 scale; Column I, p-value based on a logistic regression; Column J, -log10(p-value); Column K, bonferonni adjusted p-value.

Supplementary Data 5: Presumptive enhancers in the embryonic mouse forebrain
Combined predicted enhancers from Cicero and SnapATAC analysis (Supplementary Table 4) with ENCODE H3K27ac ChIP-Seq peaks to generate a list of high-confidence 'presumptive' enhancers in both E12.5 and E14.5 tissue. Relevant statistics and metrics: Column G, Cicero Coaccesbility Score; Column H, Per gene percentile ranking of Cicero Coaccesbility and SnapATAC -log10(p-value) after adjusting to 1-100 scale; Column I, p-value based on a logistic regression; Column J, -log10(p-value); Column K, bonferonni adjusted p-value.

Supplementary Data 6: Presumptive enhancers intersecting VISTA enhancers
List of presumptive enhancers that intersected with previously identified candidate enhancers in the VISTA dataset. Relevant statistics and metrics: Column G, Cicero Coaccesbility Score; Column H, Per gene percentile ranking of Cicero Coaccesbility and SnapATAC -log10(p-value) after adjusting to 1-100 scale; Column I, p-value based on a logistic regression; Column J, -log10(p-value); Column K, bonferonni adjusted p-value.

Supplementary Data 7: Oligonucleotides used for Capture-C
List of all oligonucleotides used to capture chromatin interactions as promoters of specific genes.
