# Supplementary material for: An epigenome atlas of neural progenitors within the embryonic mouse forebrain
Source: Nat Commun. 2022 Jul 20;13:4196. doi: 10.1038/s41467-022-31793-4 (PMC9300614; doi:10.1038/s41467-022-31793-4)
Supplement: Supplementary file 10 — Reporting Summary [file 41467_2022_31793_MOESM10_ESM.pdf]

nature research

Corresponding author(s): Timothy J. Petros
Last updated by author(s): May 26, 2021

Reporting Summary

Nature Research wishes to improve the reproducibility of the work that we publish. This form provides structure for consistency and transparency in reporting. For further information on Nature Research policies, see our Editorial Policies and the Editorial Policy Checklist.

Statistics

For all statistical analyses, confirm that the following items are present in the figure legend, table legend, main text, or Methods section.

n/a	Confirmed	
☐	☒	The exact sample size (*n*) for each experimental group/condition, given as a discrete number and unit of measurement
☐	☒	A statement on whether measurements were taken from distinct samples or whether the same sample was measured repeatedly
☐	☒	The statistical test(s) used AND whether they are one- or two-sided<br>*Only common tests should be described solely by name; describe more complex techniques in the Methods section.*
☐	☒	A description of all covariates tested
☐	☒	A description of any assumptions or corrections, such as tests of normality and adjustment for multiple comparisons
☐	☒	A full description of the statistical parameters including central tendency (e.g. means) or other basic estimates (e.g. regression coefficient) AND variation (e.g. standard deviation) or associated estimates of uncertainty (e.g. confidence intervals)
☐	☒	For null hypothesis testing, the test statistic (e.g. *F*, *t*, *r*) with confidence intervals, effect sizes, degrees of freedom and *P* value noted<br>*Give P values as exact values whenever suitable.*
☒	☐	For Bayesian analysis, information on the choice of priors and Markov chain Monte Carlo settings
☐	☒	For hierarchical and complex designs, identification of the appropriate level for tests and full reporting of outcomes
☒	☐	Estimates of effect sizes (e.g. Cohen's *d*, Pearson's *r*), indicating how they were calculated

Our web collection on statistics for biologists contains articles on many of the points above.

Software and code

Policy information about availability of computer code

Data collection	R (v4.0.0), Cellranger (v3.0.0), Cellranger-atac (v1.2.0), Seurat (v3.0.0), Signac (v1.0.0), SnapATAC (v1.0.0)
Data analysis	Monocle3 (https://cole-trapnell-lab.github.io/monocle3/), Cicero (v1.9.1), GenomicRanges (v1.42.0), DEGReport (https://github.com/lpantano/DEGreport), JASPAR (http://jaspar.genereg.net/), chromVAR (v1.12.0), Bioconductor (v3.12), Snaptools (v1.4.1), Harmony (https://github.com/immunogenomics/harmony), Capsequm (capsequm.molbiol.ox.ac.uk/cgi-bin/CapSequm.cgi), HiC-Pro (https://github.com/nservant/HiC-Pro), higlass (https://higlass.io/app), IGV (v2.12.2), Adobe Photoshop CC (20.0.9), Adobe Illustrator CC (23.1.1), Microsoft Excel (16.47.1), Python (2.7)

For manuscripts utilizing custom algorithms or software that are central to the research but not yet described in published literature, software must be made available to editors and reviewers. We strongly encourage code deposition in a community repository (e.g. GitHub). See the Nature Research guidelines for submitting code & software for further information.

Data

Policy information about availability of data

All manuscripts must include a data availability statement. This statement should provide the following information, where applicable:

- Accession codes, unique identifiers, or web links for publicly available datasets
- A list of figures that have associated raw data
- A description of any restrictions on data availability

All sequencing data (raw and processed files) generated in this study has been deposited in the Gene Expression Omnibus (GEO) database with the following accession numbers: GSE167047 (snATAC-Seq), GSE167013 (scRNA-Seq), GSE201487 (H3K4me3 CUT&Tag), GSE201488 (H3K27me3 CUT&Tag), GSE201400 (H3K27ac CUT&RUN), GSE201494 (All CUT&Tag and CUT&RUN data), GSE201186 (Hi-C) and GSE201317 (Capture-C). A searchable platform with all single cell accessibility and transcriptomic, CUT&Tag, CUT&Run, Hi-C and Capture-C data can be found on the UCSC Genome Browser:

https://www.nichd.nih.gov/research/atNICHD/Investigators/petros/epigenome-atlas.
E12.5 and E14.5 mouse forebrain H3K27ac ChIP-seq data used in this study was obtained from the ENCODE project (www.encodeproject.org), accession numbers ENCSR966AIB (E12.5) and ENCSR320EEW (E14.5). VISTA enhancers information was obtained at http://enhancer.lbl.gov.

Field-specific reporting

Please select the one below that is the best fit for your research. If you are not sure, read the appropriate sections before making your selection.

- [x] Life sciences
- [ ] Behavioural & social sciences
- [ ] Ecological, evolutionary & environmental sciences

For a reference copy of the document with all sections, see nature.com/documents/nr-reporting-summary-flat.pdf

Life sciences study design

All studies must disclose on these points even when the disclosure is negative.

Sample size

While no specific sample size calculations were performed prior to experimentation, we did have a goal for the number of cells to sequence. For each timepoint and brain region, we pooled tissue from 4-7 embryos, which was the amount of animals needed based on our preliminary experiments to obtain the desired amount of cells.
Most single cell sequencing studies perform anywhere from 1-3 replicates depending on conditions, with each replicate often encompassing several thousand cells. Since this is the first report of scATAC-seq from distinct embryonic brain regions, we wanted a minimum of 5,000 nuclei per brain region from at least 2 replicates. We obtained well over 5,000 nuclei for all samples, which was likely sufficient to identify nearly all different cell types from each region. For the scRNA-seq experiments, there are already several datasets in the literature we could for comparison, as well as unpublished ones from my lab. Thus we were confident that one replicate of > 4,000 cells would be sufficient as long as the data was in agreement with other reports and established gene expression patterns, and our data was in agreement with previous studies and know biology. For the CUT&RUN/CUT&Tag experiments, we used 100,000 nuclei for each replicate as this amount of cells was previously optimized in our hands for these reaction. For Hi-C/Capture-C experiment, we collected 1 million cells/brain region.

Data exclusions

Per standard single cell sequencing protocols, cells/nuclei that did not pass stringent QC measurements (% mitochondria reads, sufficient reads/cell, etc.) in the snATAC-seq and scRNA-seq datasets were considered outliers and excluded from analysis (as detailed in Sup figure 1). As stated in the Results section, we removed a 'mixed' cell population for analysis after Figure 2. This this population displayed properties of both GABAergic and glutamatergic neurons and, while a potentially interesting cell population, would have complicated downstream analysis that required a clean cell population from each embryonic region.

Replication

All replication details are noted in the methods section. The snATAC-seq dataset consisted of two replicates for the E12.5 MGE, LGE, CGE and cortex samples (1 cortex replicate at E12.5 and another at E14.5). The scRNA-seq datasets were one replicate each of the E12.5, CGE, MGE, LGE, cortex. Data points from the 2 replicates were highly similar (Sup fig 1), while the single replicate samples were consistent with published data sets from other labs and/or the well-characterized gene expression patterns in specific developmental ages and/or brain regions. We generated two biological reps from each brain region for the CUT&RUN (H3K27ac), CUT&Tag (H3K4me3, H3K27me3), Hi-C and Capture-C datasets. All attempts at replication were successful.

Randomization

Randomization was not applicable to tissue harvesting and sample collection because specific tissue regions were harvested from embryonic mice as desired.

Blinding

Blinding was not relevant to our study since there were no mutant vs. WT comparisons (or similar comparisons where blinding is warranted). Blinding is not necessary for all the computational analysis because all computational analysis was performed in an unbiased manner.

Reporting for specific materials, systems and methods

We require information from authors about some types of materials, experimental systems and methods used in many studies. Here, indicate whether each material, system or method listed is relevant to your study. If you are not sure if a list item applies to your research, read the appropriate section before selecting a response.

Materials & experimental systems

n/a	Involved in the study	
[ ]	[x]	Antibodies
[x]	[ ]	Eukaryotic cell lines
[x]	[ ]	Palaeontology and archaeology
[ ]	[x]	Animals and other organisms
[x]	[ ]	Human research participants
[x]	[ ]	Clinical data
[x]	[ ]	Dual use research of concern

Methods

n/a	Involved in the study	
[x]	[ ]	ChIP-seq
[x]	[ ]	Flow cytometry
[x]	[ ]	MRI-based neuroimaging

Antibodies

Antibodies used

Rabbit anti-H3K27ac (Abcam, ab4729), Rabbit anti-H3K4me3 (Active Motif, 39159), Rabbit anti-H3K27me3 (Cell Signaling, 9733T),

	Guinea Pig anti-Rabbit (Active Motif 105465)
Validation	The Active Motif H3K4me3 antibody is 'CUT&Tag validated' per the manufacturer's website (https://www.activemotif.com/catalog/1319/cut-tag-validated-antibodies). The Guinea pig anti-rabbit secondary antibody comes with the CUT&Tag IT kit, thus it is validated for CUT&Tag. The Cell Signaling H3K27me3 antibody is CUT&RUN validated per the manufacturer's website (https://www.cellsignal.co.uk/products/primary-antibodies/tri-methyl-histone-h3-lys27-c36b11-rabbit-mab/9733?N=0+102236+4294956287&Nrpp=200&No=2800&fromPage=plp). The Abcam H3K27ac antibody is ChIP-grade per the manufacturer's website (https://www.abcam.com/histone-h3-acetyl-k27-antibody-chip-grade-ab4729.html), and has been used in many papers for CUT&Tag (e.g., https://www.sciencedirect.com/science/article/pii/S2666166721008066).

Animals and other organisms

Policy information about studies involving animals; ARRIVE guidelines recommended for reporting animal research

Laboratory animals	All experimental procedures were conducted in accordance with the National Institutes of Health guidelines and were approved by the Eunice Kennedy Shriver NICHD Animal Care and Use Committee (protocol #20-047). The following mouse lines were used in this study: C57BL/6J (JAX# 000664). For timed matings, noon on the day a vaginal plug was observed was denoted E0.5. For each experimental modality, brain regions from multiple embryos (> 5) was pooled together prior to single cell dissociations. Both male and female embryonic mice were used without bias for all experiments. Housing conditions: 12/12 hour light/dark cycle, humidity between 30-50%, temperature 72 degrees C.
Wild animals	No wild animals were used in this study.
Field-collected samples	No field collected samples were used in this study.
Ethics oversight	All experimental procedures were conducted in accordance with the National Institutes of Health guidelines and were approved by the NICHD Animal Care and Use Committee (ACUC), Animal Study Protocol# 20-047.

Note that full information on the approval of the study protocol must also be provided in the manuscript.
